# Mass spectrometric quantitation of covalently bound cell wall proteins in *Saccharomyces cerevisiae*

**DOI:** 10.1111/j.1567-1364.2007.00272.x

**Published:** 2007-07-06

**Authors:** Qing Yuan Yin, Piet W J de Groot, Luitzen de Jong, Frans M Klis, Chris G De Koster

**Affiliations:** 1 Laboratory for Biomacromolecular Mass Spectrometry; 2 Molecular Biology and Microbial Food Safety, Swammerdam Institute for Life Sciences, University of Amsterdam WV Amsterdam, The Netherlands

**Keywords:** cell wall protein, proteomics, MS, glycosylphosphatidylinositol-anchored protein, pir protein, transglycosylase

## Abstract

The cell wall of yeast consists of an internal skeletal layer and an external layer of glycoproteins covalently linked to the stress-bearing polysaccharides. The cell wall protein (CWP) population consists of over 20 different proteins, and may vary in composition. We present two complementary methods for quantifying CWPs, based on isobaric tagging and tandem MS: (1) absolute quantitation of individual CWPs, allowing estimation of surface densities; and (2) relative quantitation of CWPs, allowing monitoring of the dynamics of the CWP population. For absolute quantitation, we selected a representative group of five proteins (Cwp1p, Crh1p, Scw4p, Gas1p, and Ecm33p), which had 67 × 10^3^, 44 × 10^3^, 38 × 10^3^, 11 × 10^3^ and 6.5 × 10^3^ of wall-bound copies per cell, respectively. As Cwp1p is predominantly incorporated in the birth scar, this corresponds to a protein density of *c*. 22 × 10^3^ copies μm^−2^. For relative quantitation, we compared wild-type cells to *gas1*Δ cells, in which the cell wall integrity pathway is constitutively activated. The levels of Crh1p, Crh2p, Ecm33p, Gas5p, Pst1p and Pir3p increased about three- to fivefold, whereas the level of Scw4p was significantly decreased. We propose that our methods are widely applicable to other fungi.

## Introduction

Fungal cells are surrounded by a sturdy wall, enabling them to withstand the internal osmotic pressure, and providing protection against mechanical injury. The fungal wall is also instrumental in adhesion to abiotic and biotic surfaces and in pathogenesis. The cell wall of *Saccharomyces cerevisiae* has two layers: an internal layer, which consists of a network of branched β-1,3-glucan molecules extended with covalently attached β-1,6-glucan and chitin molecules, and an outer layer, which is mainly composed of mannoproteins covalently linked to the underlying glycans (Klis *et al.*, 2006). A similar cell wall structure is also found in other ascomycetous yeasts, such as the human pathogens *Candida albicans*, *C. glabrata*, *Exophiala dermatitidis* and *Sporothrix schenckii*, in the fission yeast *Schizosaccharomyces pombe*, and to a lesser extent in mycelial ascomycetous fungi (Travassos, 1985; Brul *et al.*, 1997; Montijn *et al.*, 1997; De Groot *et al.*, 2004, De Groot 2007; Weig *et al.*, 2004; Damveld *et al.*, 2005; Klis *et al.*, 2006).

In the external protein layer of baker's yeast, at least 20 different types of cell wall proteins (CWPs) have been identified (Protchenko *et al.*, 2001; Yin *et al.*, 2005; Verstrepen & Klis, 2006). Most of them are glycosylphosphatidylinositol (GPI)-modified and are therefore called GPI-CWPs. GPI-CWPs include flocculins, adhesins, transglycosidases, proteins involved in iron utilization, and proteins of unknown function (Klis *et al.*, 2006). A smaller group of CWPs are directly linked to the β-1,3-glucan network through an alkali-sensitive linkage (ASL), including the family of Pir-CWPs (Pir, proteins with internal repeats) (Mrsa *et al.*, 1997). Pir-CWPs may be involved in crosslinking the β-1,3-glucan network through their repeats, presumably by the formation of a carboxyl ester bond between a specific glutamine residue and a glucosyl hydroxyl group (Ecker *et al.*, 2006). The composition of the cell wall proteome is highly dynamic, and depends on growth conditions such as nutrient availability, temperature, external pH, and oxygen level (Klis *et al.*, 2006). Furthermore, the expression and incorporation into the cell wall of CWPs is temporally and spatially controlled. When the cell wall is stressed, either constitutively due to a genetic defect, or temporarily and dose-dependently by growing the cells in the presence of cell wall-perturbing compounds, the cells tend to become swollen, as their walls are less capable of withstanding the turgor pressure. In response to cell wall stress, the cell wall integrity pathway is activated, resulting in increased expression of a specific set of CWPs, massive deposition of chitin in the lateral walls, and much higher levels of the CWP–polysaccharide complex CWP→β-1,6-glucan←chitin (Kapteyn *et al.*, 1997; Jung & Levin, 1999; Lagorce *et al.*, 2003; Levin, 2005). A representative cell wall mutant is *gas1*Δ, in which a gene encoding a β-1,3-glucan remodeling enzyme is deleted (Popolo & Vai, 1999).

Quantitation of CWPs by gel-based methods is notoriously difficult, because CWPs are heavily glycosylated and often also phosphorylated. They occur as mixtures of multiple glycoforms and phosphoforms, resulting in multiple fuzzy spots per protein (Weig *et al.*, 2004). In this study, we used isobaric tagging and tandem MS (MS/MS) for absolute and relative quantitation of CWPs. Our method is gel-independent and does not require a specific antiserum or a tagged form of the target proteins. In addition, as the number of fully sequenced fungal genomes is rapidly growing, and because many fungi, such as *Candida* spp., *Sc. pombe*, *Aspergillus niger*, *Fusarium oxysporum* and *Ustilago maydis*, and also the oomycete *Phytophthora ramorum*, possess covalently bound CWPs, our methods will be widely applicable (Ruiz-Herrera *et al.*, 1996; Schoffelmeer *et al.*, 1996, Schoffelmeer 2001; Brul *et al.*, 1997; Weig *et al.*, 2004; Damveld *et al.*, 2005; Meijer *et al.*, 2006; De Groot *et al.*, 2007).

## Materials and methods

### Yeast strains and media

Cells from the wild-type strain FY833 (*MAT*a *his3*Δ*200 ura3-52 leu2*Δ*1 lys2*Δ*202 trp1*Δ*63*), and the mutant strain AR104 (FY833 *gas1::LEU2*), were grown to exponential phase at 30°C in YPD medium (1% yeast extract, 2% Bacto peptone and 2% glucose). At harvest, the OD_600 nm_ was 1.2; OD_600 nm_=1.0 corresponds to 1.66±0.05 × 10^7^ cells mL^−1^ (mean±SD, *n*=3) as measured with a Multisizer Coulter Counter (Beckman Coulter, Fullerton, CA).

### Cell wall isolation and protein content determination

Cell walls were isolated as described by De Groot *et al.* (2004). The cell wall protein content was determined with the BCA protein assay (Perbio Science, Erembodegem, Belgium) as described by Kapteyn *et al.* (1997).

### Cell wall digestion

Sodium dodecyl sulfate-extracted cell walls were reduced, alkylated, and freeze-dried overnight, as previously described (Yin *et al.*, 2005). Freeze-dried cell walls (4±0.1 mg; mean±SD, *n*=3) were resuspended in 50 μL of 0.5 M triethylammonium bicarbonate, pH 8.5, and incubated overnight at 37°C with sequencing-grade trypsin (Roche Applied Science, Mannheim, Germany), using a protein/enzyme ratio of 50 : 1. This procedure has been immunologically shown to result in a complete loss of the signal in the case of Cwp1p, Pir2p, and Ccw14p, indicating that tryptic digestion is exhaustive (Yin *et al.*, 2005). Digested samples were centrifuged, and the supernatants containing the solubilized peptides were completely dried in a vacuum concentrator.

### Preparation of defined standard solutions of labeled peptides

The synthetic peptides WFTDLK(68–73) (for Crh1p), SSSGFYIK(106–114) (for Cwp1p), VIDGFNK(285–291) (for Ecm33p), TAEFK(248–252) (for Gas1p) and WLLEQIQR(301–308) (for Scw4p) were purchased from KJ Ross-Petersen ApS (Klampenborg, Denmark). The sequence identity of the peptides was verified by amino acid analysis and confirmed by MS. Synthetic peptides, 0.4–0.7 mg, were dissolved in 10 μL of 0.5 M triethylammonium bicarbonate, pH 8.5. Five microliters of iTRAQ_114_ reagent (Applied Biosystem, Foster City, CA) was added, and the mixture was incubated at room temperature for 1 h. The iTRAQ_114_-labeled synthetic peptides were diluted 100- to 1000-fold, and were used as stock solutions. The concentrations of the standard peptides in stock solutions were determined spectrophotometrically on the basis of the molar extinction coefficients of phenylalanine, tryptophan or tyrosine residues (195, 5579, and 1405 M^−1^ cm^−1^, respectively) (Fasman, 1976). The UV absorbances at 257.5 nm (for peptides TAEFK and VIDGFNK), 280 nm (for peptides WFTDLK and WLLEQIQR) and 274 nm (for peptide SSSGFYIK) were used to calculate peptide concentrations. Stock solutions of the five peptides were mixed to obtain a working standard solution, in which the concentration of each peptide was normalized to 0.32–0.54 μM.

### Isobaric labeling of cell wall peptides and synthetic peptides

**Fig. 1:**
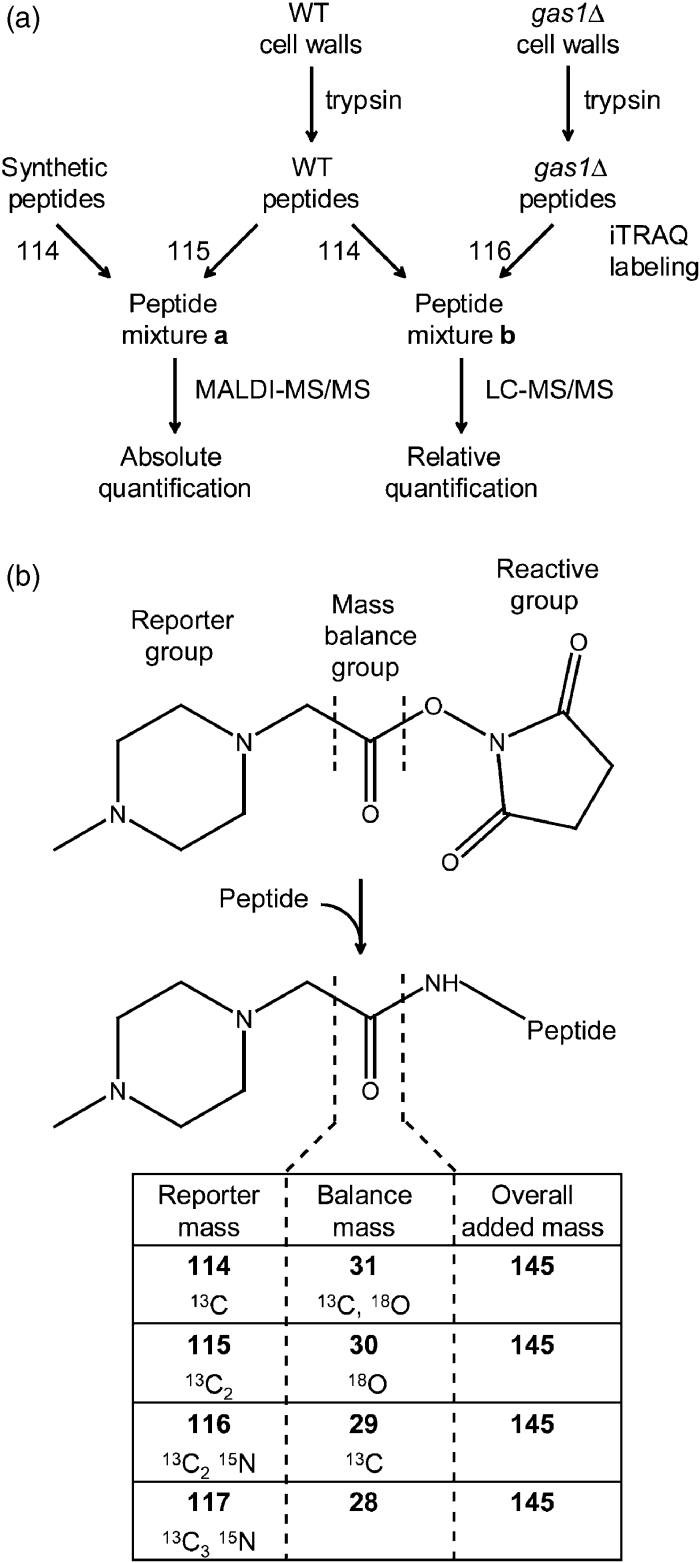
(a) Experimental steps of absolute and relative quantitation of cell wall proteins using iTRAQ. For absolute quantitation, wild-type cell wall peptides were labeled with iTRAQ_115_ reagent and then mixed with iTRAQ_114_-labeled synthetic peptides. The peptide mixture a was subsequently subjected to MALDI-MS/MS. For relative quantitation, equal amounts of dry cell walls from wild-type and *gas1*Δ cells were digested with trypsin. The resulting peptides were labeled with iTRAQ_114_ and iTRAQ_116_, respectively. The experiment was repeated on samples from a replicate culture with iTRAQ_117_ for the wild-type sample and iTRAQ_114_ for the *gas1*Δ sample (see ‘Materials and methods’). The peptide mixture b was subsequently subjected to LC-MS/MS. (b) A diagram of the iTRAQ reaction. Each iTRAQ reagent molecule consists of a reporter group (based on *N*-methylpiperazine), a mass balance group (the carbonyl group), and a peptide-reactive group (*N*-hydroxylsuccinimide ester). The overall mass of reporter and balance components of the molecule is kept constant using differential isotopic enrichment with ^13^C, ^15^N and ^18^O atoms. Peptides from differently labeled samples are therefore chromatographically indistinguishable and isobaric, but yield specific reporter ions at *m*/*z* 114, 115, 116 or 117 following MS/MS.

Absolute and relative amounts of CWPs were determined using the iTRAQ methodology (Ross *et al.*, 2004). Figure 1a presents an overview of the essential steps of the quantitation experiments. In brief, peptide samples were incubated with one or more of the four available iTRAQ reagents (Applied Biosystem) to derivatize the N-termini and the lysine residues of the peptides. Each iTRAQ reagent molecule consists of a reporter group (based on *N*-methylpiperazine), a mass balance group (the carbonyl group), and a peptide-reactive group (*N*-hydroxylsuccinimide ester) (Fig. 1b). The overall added mass of reporter and balance components of the molecule is kept constant so that derivatized peptides from different samples are isobaric (i.e. of the same mass) and chromatographically indistinguishable, but yield specific reporter ions at *m*/*z* 114, 115, 116 or 117 following collision-induced dissociation in a mass spectrometer. The relative concentrations of the peptides are thus deduced from the relative intensities of the corresponding reporter ions.

For absolute quantitation, dried peptides from 2 mg of cell walls were resuspended in 20 μL of acetonitrile and 10 μL of 0.5 M triethylammonium bicarbonate, pH 8.0. Ten microliters of iTRAQ_115_ reagent was added, and the reaction mixture was incubated at room temperature for 1 h. The labeled peptides were dried to remove organic solvent, and resuspended in 10 μL of H_2_O. Subsequently, the labeled cell wall peptides were mixed with the working standard solution of the synthetic peptides at ratios of 1 : 5, 1 : 1 and 5 : 1 (v:v). For relative quantitation, equal amounts of dried cell walls from wild-type and *gas1*Δcells were digested with the endoprotease trypsin. The resulting peptides were labeled with iTRAQ reagents (iTRAQ_114_ on the wild-type sample and iTRAQ_116_ on the *gas1*Δ sample). The experiment was repeated on samples from a replicate culture with a different combination of iTRAQ tags, i.e. iTRAQ_117_ for the wild-type sample, and iTRAQ_114_ for the *gas1*Δ sample.

### Matrix-assisted laser-desorption ionization (MALDI)-MS/MS analysis

For absolute quantification, peptides were collected on Ziptip μC_18_ pipette tips (Millipore, Bedford, MA), washed with 0.1% formic acid, and eluted with 60% acetonitrile/0.1% formic acid. Subsequently, 0.5 μL of the eluate was mixed with 0.5 μL of α-hydroxy cinnaminic acid (10 μg μL^−1^ in acetonitrile/ethanol 1 : 1, v:v). The mixture was spotted on a MALDI target plate and allowed to dry. MS was performed using a QSTAR XL Hybrid LC/MS/MS system (Applied Biosystems, Framingham, MA) with its oMALDI (orthogonal MALDI) ion source. To obtain precursor masses of target peptides, the mass spectrometer was set to perform data acquisition in the positive ion mode, with a selected mass range of *m*/*z*800–2000. Peptides were selected for MS/MS, and the time of summation of MS/MS events was set at 3 s.

### Liquid chromatography (LC)-MS/MS analysis

For relative quantification, six LC-MS/MS runs were performed for each sample of mixed peptides from wild-type and *gas1*Δ cells. Proteolytic digests derived from 30 μg of freeze-dried cell walls were fractionated on a 150 mm × 75 μm (length × inner diameter) reversed-phase capillary column (PepMap C18, Dionex). Sample introduction and mobile phase delivery at 300 nL min^−1^ were performed using an Ultimate nano-LC system (Dionex, Sunnyvale, CA) equipped with a 10-μL injection loop. Mobile phase A was water+0.1% formic acid, and mobile phase B was 80% acetonitrile+0.1% formic acid. For the separation of peptides, a linear gradient of 5–95% B over 30 min was employed. Eluting peptides were directly electrosprayed into a Micromass quadrupole time-of-flight mass spectrometer (Waters, Milford, MA). The most abundant ions from the survey spectrum, ranging from *m*/*z*350 to 2000, were automatically selected for collision-induced fragmentation using masslynx software. Fragmentation was conducted with argon as collision gas at a pressure of 4 × 10^−5^ bars as measured on the quadrupole pressure gauge. Resulting MS/MS spectra were processed with the maxent3 algorithm embedded in masslynx software to generate peak lists.

### Data analysis

Mass spectra were transformed and submitted to mascot (version 2.05, Matrix Science, London, UK) by the script provided in the data acquisition and analysis software analyst qs (Applied Biosystems), or by the peak list (pkl) file generated from the software masslynx (Waters). Each MS/MS spectrum was searched against an in-house database (Yin *et al.*, 2005), resulting in a set of tryptic peptide matches with confidence values. These peptide identifications were then combined using the mascot search engine to yield a set of cell wall protein identifications with confidence values. The mascot searches were run using the following parameters: fixed carbamidomethyl modification of cysteine residues; fixed iTRAQ modification of free amine in the N-terminus and lysine; and variable methionine oxidation, and glutamine and asparagine deamidation. Incomplete tryptic digestion with one missed cleavage was allowed; precursor error tolerance was set at ±0.8 Da; MS/MS fragment tolerance was set at ±0.4 Da; and charge was set at+1. Furthermore, only peptides matching the identified protein better than any other protein in the database (first rank) with *P*<0.05 for random occurrence were retained for further analysis. For each MS/MS spectrum acquired, signature ion peak areas at *m*/*z*114.1, 115.1, 116.1 and 117.1 were extracted using analyst qs or masslynx software.

For absolute quantitation, in the three mixing experiments, MS/MS spectra that showed the closest signal-to-noise ratios at the signature ion region were selected for quantitation. For relative quantitation, the ratios of the raw peak heights were normalized against the amount of dried cell walls used. Because the accuracy of ion intensity measurement may be affected by its signal-to-noise ratio in the time-of-flight type of mass spectrometer used in this study, spectra with the intensity of reporter ion peaks below a predefined threshold were considered as noise and were filtered using masslynx software. The remaining spectra were used to calculate the mean peptide ratios and SDs. We took into consideration that each protein can potentially be identified by a number of peptides and every peptide can be measured multiple times. Finally, protein ratios from the duplicate labeling experiments (iTRAQ_114_ vs. iTRAQ_116_, and iTRAQ_117_ vs. iTRAQ_114_) were averaged. For proteins quantified with only two peptides, the raw data were manually inspected, and a protein ratio was calculated only if the two peptide ratios differed by no more than 25%.

## Results and discussion

### Cellular quantitation of covalently linked cell wall proteins

Previously, we identified 19 covalently bound CWPs of *Sa. cerevisiae*, seven of which (Crh1p, Crh2p, Gas1p, Gas3p, Gas5p, Scw4p, and Scw10p) were classified as glycoside hydrolases (Henrissat & Davies, 1997). Phenotypic analysis of deletion mutants lacking the corresponding encoding genes indicated that most of them have altered cell wall properties, which reinforces the importance of the identified proteins for proper cell wall formation. These studies, however, did not provide quantitative information on the CWPs identified. To address this question, we determined as a first step the total number of CWP molecules in a log-phase haploid cell. Yeast cells, 5 × 10^8^ (harvested from 25 mL of medium), were freeze-dried, yielding a dry weight of 9.3±0.2 mg (mean±SD, *n*=3), which corresponds to 18.6±0.4 pg (mean±SD, *n*=3) biomass per cell. In parallel, the same number of cells was used to isolate cell walls as previously described (De Groot *et al.*, 2004), showing that the cell wall dry weight accounted for *c*. 21% of the total biomass (3.9±0.3 pg cell^−1^; mean±SD, *n*=3). This is in agreement with a previous report showing that in rich medium the cell wall accounts for *c*. 20% of the dry biomass per cell (Aguilar-Uscanga & François, 2003). The peptide parts of the covalently bound CWPs were determined using the BCA protein assay to be 4% of the wall biomass, which corresponds to 0.16 pg cell^−1^, consistent with a previous report (Kapteyn *et al.*, 2001). Assuming a mean molecular mass of CWPs of 50 kDa, a haploid cell contains 1.9 × 10^6^ covalently attached CWP molecules.

As CWPs seem to be uniformly distributed over the cell surface (Tokunaga *et al.*, 1986; Baba & Osumi, 1987), it is possible to estimate their surface density by calculating the cell surface of an average yeast cell. Yeast cells were assumed to approximate two prolate spheroids (the mother cell and the bud cell) in conjunction with each other. The surface area *S* was calculated from the semi-major (*a*) and semi-minor (*b*) axes and the equation *S*=2*π*(*b*^2^+*abœ*/sin*œ*), where *œ*=arccos(*a/b*). The values for the major and minor axes of the adult cell (4.57 and 3.82 μm, respectively) and for the major and minor axes of the bud (1.85 and 1.70 μm, respectively) were averaged from the data of 126 wild-type strains in *Sa. cerevisiae* Morphological Database (SCMD, http://scmd.gi.k.u-tokyo.ac.jp). The surface area *S* was calculated to be *c*. 60 μm^2^, which corresponds to an average CWP density in the cell wall of *c*. 31 × 10^3^ molecules μm^−2^. Considering that a spherical protein such as hemoglobin (*M*_r_ 64 500), which has a diameter of about 5.5 nm (Lehninger *et al.*, 1993), needs about 24 nm^2^ to accommodate it on a surface (corresponding to *c*. 38 × 10^3^ copies μm^−2^ in hexagonal packing; http://mathworld.wolfram.com/CirclePacking.html), these results suggest that CWPs are closely packed on the cell surface, as also suggested by ultrastructural studies (Tokunaga *et al.*, 1986; Baba & Osumi, 1987) and consistent with their being largely present in the form of a monolayer. It has to be noted that CWPs are frequently heavily glycosylated and may carry extended N-chains of up to 200 sugar residues (Orlean, 1997) and thus occupy more space than their protein parts alone. This predicted dense packing of CWPs on the cell surface is also consistent with the observation that the N-chains of CWPs have been shown to limit the permeability of the yeast cell wall for macromolecules (De Nobel *et al.*, 1990).

### Absolute quantitation of five covalently linked cell wall proteins

In a recent genome-wide study using fusion constructs with a C-terminally attached green fluorescent protein (GFP), the absolute quantity of yeast proteins was determined (Ghaemmaghami *et al.*, 2003). Unfortunately, the analysis of CWPs using GFP fusion constructs did not take into account that the C-terminal hydrophobic part of GPI-modified proteins is removed in the endoplasmic reticulum by a transamidase complex (Ohishi *et al.*, 2000). Their estimates do not, therefore, reflect the protein amount at the cell surface, and are probably too low. For example, a yeast cell was estimated to contain only *c*. 2000 copies of Cwp1p (*Saccharomyces* Genome Database; Ghaemmaghami *et al.*, 2003). We therefore developed an alternative approach for CWP quantitation based on using defined concentrations of synthetic peptides, isobaric tagging with the iTRAQ reagent, and tandem MS (Ross *et al.*, 2004). To this end, five CWPs were selected to determine their absolute quantities, including a member from each of three carbohydrate-active enzyme families (the two GPI-dependent CWPs Crh1p and Gas1p, and the ASL-CWP Scw4p) found in the cell wall, Ecm33p, another GPI-dependent CWP of unknown function, which has been postulated to be a carbohydrate-active enzyme as well (Klis *et al.*, 2006), and the structural CWP Cwp1p.

To develop a suitable internal standard, one peptide from each target protein was chosen on the basis of its amino acid sequence and the cleaving characteristics of the protease trypsin. The selection criteria we used were as follows: (1) the peptides were observed in previous MS/MS experiments, indicating that they are sensitive to the ionization process; (2) the peptides do not contain methionine or cysteine residues, so that they are stable under oxidative conditions; (3) the peptides contain as few serine and threonine residues as possible, as these may undergo a side reaction with the iTRAQ reagent; and (4) the peptides must be unique for the corresponding protein but not too large to complicate synthesis and analysis. Five peptides (Table 1) were thus order-synthesized, derivatized with an isobaric iTRAQ tag, and evaluated by MALDI-MS/MS. This evaluation process provided qualitative information about peptide ionization efficiency in MS collision-induced fragmentation, and about their identity (data not shown).

**Table 1:** Absolute quantitation of covalently linked cell wall proteins

Protein	Synthetic peptide	Residue numbers	Peptide mass	Observed mass*	Copies cell^−1^× 10^−3^
GPI protein					
Crh1p	WFTDLK	68–73	808.41	1097.63	44
Cwp1p	SSSGFYAIK	106–114	958.48	1247.70	67
Ecm33p	VIDGFNK	285–291	791.42	1080.64	6.5
Gas1p	TAEFK	248–252	594.30	883.52	11
ASL protein					
Scw4p	WLLEQIQR	301–308	1084.60	1229.71	38

**Fig. 2:**
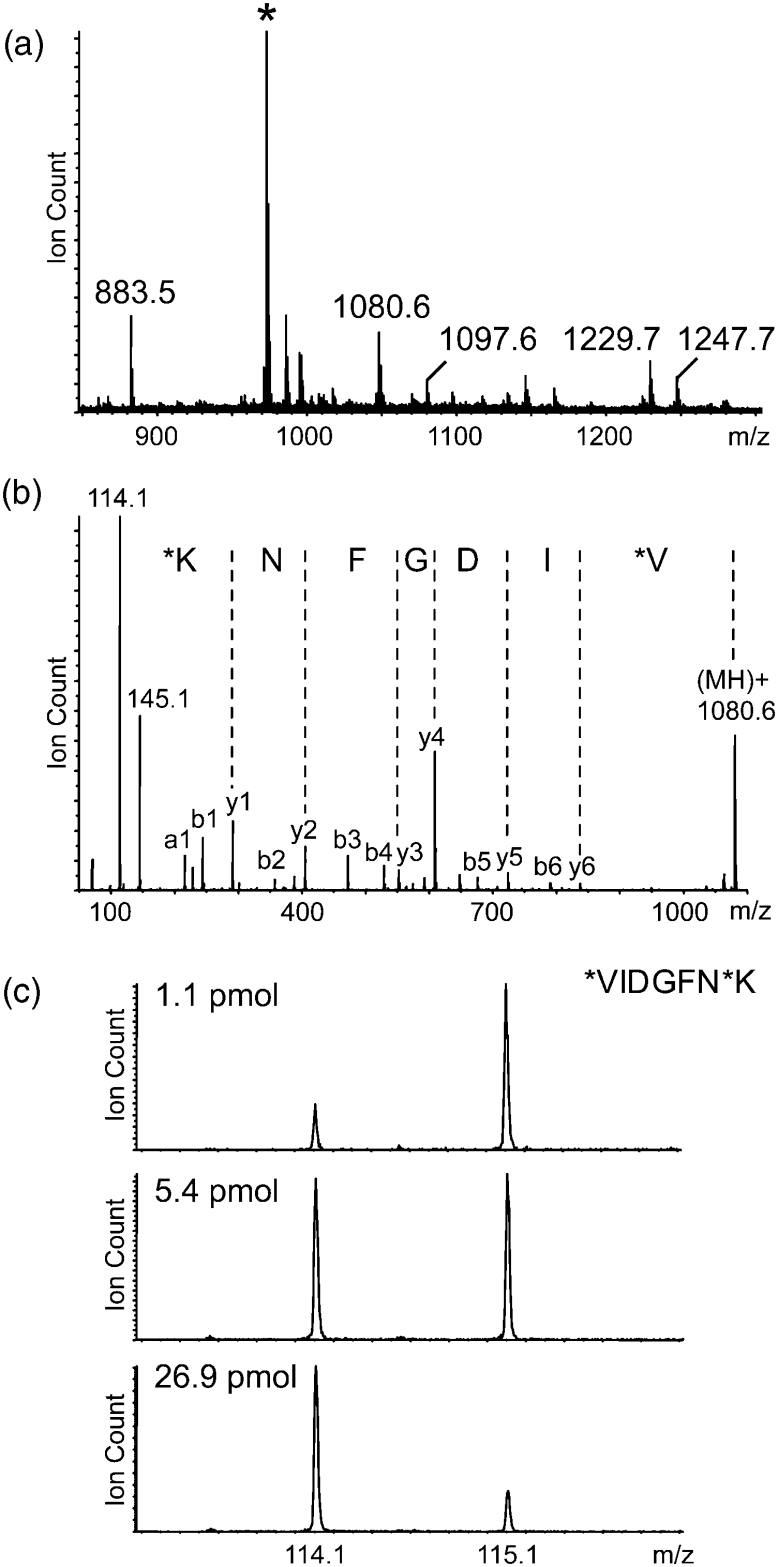
MS and MS/MS spectra of peptides from a tryptic cell wall protein digest titrated with synthetic peptides. (a) Overview of a MALDI-MS spectrum showing the total cell wall protein digest mixed with labeled synthetic peptides (peptide mixture a in Fig. 1). The base peak labeled with an asterisk is from a shared peptide of Pir proteins (peptide b in Fig. 3). The five indicated precursor ions were fragmented to obtain quantitative information. (b) The MS/MS spectrum of the precursor ion 1080.6 [M+H]^+^ corresponding to the peptide ^*^VIDGFN^*^K from Ecm33p (^*^denotes the labeled amino groups). The sequence was derived from both b-ion and y-ion series, confirming the identity of the peptide. (c) Expanded view of the signature ion region of three MS/MS spectra of Ecm33p peptide VIDGFNK from 5 × 10^8^ cells (peaks at 115.1) titrated with (from top to bottom) 1.08, 5.4 and 27 pmol of synthetic peptide (peaks at 114.1), allowing an accurate estimation of the absolute quantity of the peptide studied.

* Observed masses ([M+H]^+^) of iTRAQ-labeled peptides in the MS spectrum (Fig. 2a). The iTRAQ reagent labels the N-termini and the ɛ-amino group from lysine residues of peptides. Peptide identities were confirmed by MS/MS before and after the labeling.

GPI, glycosylphosphatidylinositol; ASL, alkali-sensitive linkage.

When titrated with a defined amount of the synthetic peptide, an iTRAQ-labeled tryptic peptide from natural CWP is chromatographically indistinguishable from the internal standard peptide and has an identical mass. Therefore, in a typical MALDI-MS spectrum of CWPs (Fig. 2a), the subsequent MALDI-MS/MS analysis provides a highly specific and sensitive measurement of both the internal standard and the analyte, directly from a mixture of hundreds of cell wall peptides. In this MS spectrum, peaks of interest (i.e. synthetic peptides and their native counterparts from target CWPs) could be easily recognized on the basis of their *m*/*z* value (Fig. 2a, mass-labeled). Taking the peptide ^*^VIDGFN^*^K of Ecm33p (^*^denotes the labeled amino groups) as an example, this particular peptide has two labeling sites, resulting in an observed precursor *m*/*z* of 1080.6. The precursor ion could be sent to fragmentation for sequence verification and for quantification. Figure 2b shows a representative MS/MS spectrum. The series of both y and b product ions from MS/MS and the precursor ion *m*/*z* have been used to verify the identity of this peptide. The isobaric tags from the iTRAQ label produced abundant MS/MS signature ions of *m*/*z*114.1 and 115.1, representing the ion of the N-methylpiperazinylated N-terminus. The stability of the resulting alkylated immonium ion is universal for essentially all modified tryptic peptides, as it is independent of the amino acid at the N-terminus of the peptide. The relative intensities of the signature ions correspond to the proportions of the synthetic and natural peptides. Figure 2c zooms in on the reporter region of the MS/MS spectra of the peptide VIDGFNK derived from 5 × 10^8^ yeast cells that were titrated with 1.1, 5.4 and 26.9 pmol of standard peptides (from top to bottom, respectively). The peaks in the middle spectrum showed the closest signal-to-noise ratio, and the ratio of the peak areas at *m*/*z* 114.1 and 115.1 was then used to determine the amount (5.4 pmol) of this peptide in the original cell wall material, resulting in an estimated 6.5 × 10^3^ wall-bound Ecm33p molecules per cell (Table 1). The absolute quantitation of all five CWPs is summarized in Table 1.

**Fig. 3:**
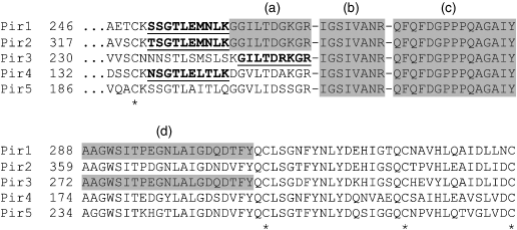
MS identification of the members of the Pir family. Only the C-terminal regions of the proteins are depicted. The numbers correspond to the positions of amino acid residues in the ORFs of each Pir protein. Identification and quantitation cannot be realized on the basis of the tryptic peptides b and c, which occur in all members of the Pir family, or on a and d, which are shared by two members (highlighted). Pir1p–Pir4p contain a unique tryptic peptide (bold and underlined). A unique tryptic peptide from Pir5p or tryptic peptides from the region preceding the conserved four-cysteine (indicated by asterisks) domain have never been observed. Owing to trypsin miscleavage, peptide a appears in two forms: GGILTDGK and GGILTDGKGR. Peptides c and d are generated due to aspecific enzymatic activity of trypsin (chymotrypsin).

Our experimental results are in reasonable agreement with the notion that dividing haploid cells contain on average 1.9 × 10^6^ covalently attached CWPs per cell (see above), considering that there are over 20 CWPs incorporated in the wall of a log-phase-grown cell. Cwp1p is the most abundant cell wall protein observed in this study; the amount of 67 × 10^3^ per cell is indeed much higher than previously reported by Ghaemmaghami *et al.* (2003). Assuming that Cwp1p is preferentially incorporated in the birth scar area (Smits *et al.*, 2006), and using an estimated birth scar area of *c*. 3 μm^2^ (measured from Fig. 2, Smits *et al.*, 2006), this would correspond to a local density of Cwp1p of *c*. 22 × 10^3^ copies μm^−2^. As the average CWP density of the cell wall is *c*. 31 × 10^3^ molecules μm^−2^, this suggests that Cwp1p is the major CWP found in this area. Interestingly, Cwp1 can function as a crosslinking protein, because it is one of the few GPI-CWPs that also possesses a Pir repeat, which may be used to couple it directly to β-1,3-glucan in addition to its linkage to β-1,6-glucan (Kapteyn *et al.*, 2001). This suggests that it may protect the birth scar against cell wall damage during cytokinesis. Wall-bound Crh1p is present at 44 × 10^3^ copies per cell. As its incorporation in the wall is cell cycle-dependent, and as it is only found at the emerging bud site and, later in the cell cycle, in the neck region, its local density is considerable, consistent with its reported function in interconnecting chitin to β-glucan (Rodríquez-Peña *et al.*, 2000; Cabib *et al.*, 2007). The low copy numbers per cell of the wall-bound forms of Gas1p and Ecm33p are consistent with the empirical and theoretical evidence that Gas1p and Ecm33p are mainly targeted to the plasma membrane (Conzelmann *et al.*, 1988; Caro *et al.*, 1997; Hamada *et al.*, 1999). The ASL-CWP Scw4p seems to be moderately expressed in the cell wall.

### Relative quantitation of the cell wall proteome

To examine the changes in the CWP population as a result of cell wall stress, we performed a quantitative proteomics study comparing wild-type and *gas1*Δ cells. In total, 367 peptides were identified, of which 113 were suitable for quantitation (Table 2). Fourteen cell wall proteins were unambiguously identified, and their abundance ratios (*gas1*Δ cells vs. wild-type cells) were quantified. Although most of the abundance ratios of covalently bound proteins were derived from multiple observations, this was not the case for proteins from Pir family. The members of this protein family are highly homologous, and all contain a variable number of glutamine-containing internal repeats and a C-terminal domain containing four cysteine residues with fixed spacing (Fig. 3). The internal repeat domain is highly mannosylated, and is therefore not identified by our approach. Only the C-terminal regions of these proteins provide suitable tryptic peptides for identification and quantitation. However, as this region is highly conserved, the sequences of four frequently identified peptides (Fig. 3a–d) are (partially) shared between the members of the Pir family. Therefore, unambiguous identification and quantitation of individual Pir proteins can only be based on those peptides that are unique to each individual protein (Fig. 3, bold and underlined). This resulted in a relatively low frequency of observations of unique peptides from Pir1p and Pir3p. It has to be noted that none of the identified peptides in this study is unique to the fifth member of this family (Pir5/YJL160C). Therefore, its presence cannot be verified. Finally, in a previous study, we identified six other covalently linked CWPs in exponentially growing yeast cells (Yin *et al.*, 2005); two of them could not be identified by trypsin digestion, but were instead identified using the endoprotease Glu-C and are thus missed here. The remaining four CWPs are relatively rare, and might have been missed because of the lower sensitivity inherent in the approach taken in the current study.

**Table 2:** Relative quantitation of covalently linked cell wall proteins in WT and *gas1*Δ cells

Protein	Number of quantified peptides	Protein ratio ± SD*	mRNA ratio†	Function and properties‡
GPI protein‡				
Crh1p	15	4.65 ± 0.65	3.84	GH16, transglycosylase crosslinking chitin to β-1,6-glucan
Crh2p	6	3.58 ± 0.59	0.86	GH16, transglycosylase crosslinking chitin to β-1,6-glucan
Cwp1p	24	2.95 ± 0.79	1.10	Contains a Pir-like repeat
Ecm33p	6	4.76 ± 1.39	1.15	Related to cell wall biogenesis
Gas1p§	4	0.18 ± 0.05	0.04	GH72, transglucosidase extending β-1,3-glucan
Gas3p	6	0.99 ± 0.26	0.81	GH72
Gas5p	2	3.00 ± 0.61	1.42	GH72, transglucosidase
Pst1p	17	5.33 ± 1.74	3.87	Related to cell wall biogenesis
Ssr1p	7	1.42 ± 0.25	0.83	CFEM domain
ASL protein‡				
Pir1p	1	1.09	3.26	Conserved 4-C domain
Pir2p	8	2.98 ± 0.80	2.30	Conserved 4-C domain
Pir3p	2	3.17 ± 0.34	6.64	Conserved 4-C domain
Pir4p	7	2.09 ± 0.27	3.42	Conserved 4-C domain
Scw4p	8	0.50 ± 0.08	0.78	GH17, β-1,3-glucanase

* Protein ratios (*gas1*Δ/wild type) are means of peptide ratios from two duplicated labeling experiments.

† mRNA data were obtained from http://www.dkfz.de/funct_genome/yeast-data.html.

‡ GPI, glycosylphosphatidylinositol; ASL, alkali-sensitive linkage; GH, glycoside hydrolases, classification according to Henrissat & Davies (1997); CFEM, common in fungal extracellular membrane/wall proteins.

§ Gas1p data in *gas1*Δ cells represent the background noise of MS experiments.

The protein level of isolated walls of *gas1*Δ cells is about twice as high as in wild-type cell walls, consistent with the known constitutive activation of the cell wall integrity pathway in these cells (7.7% vs. 4.0%, own observation; Kapteyn *et al.*, 1997). This implies that, without any changes in the relative protein composition in the wall, the abundance ratios should average about two. The abundance ratios in Table 2 indicate that activation of the cell wall integrity pathway as a result of the cell wall defects in *gas1*Δ cells triggers a considerable increase in the absolute cell wall levels of five putative carbohydrate-active enzymes (Crh1p, Crh2p, Gas5p, and also Ecm33p and Pst1p), and four predicted structural CWPs (Cwp1p, Pir2p, Pir3p, and Pir4p), whereas the level of Scw4p is strongly decreased. For comparison, we have included the corresponding mRNA ratios obtained by microarray analysis of *gas1*Δ and wild-type cells (Lagorce *et al.*, 2003). Interestingly, the ratios of the wall-bound forms of the ASL proteins tend to be lower than the corresponding transcript ratios. As these proteins, at least partially, may end up in the medium (Russo *et al.*, 1992; Kapteyn *et al.*, 2001), this raises the question of whether, in *gas1*Δ cells ASL proteins are less efficiently incorporated into the walls. Conversely, the protein ratios of Crh2p and Ecm33p are considerably higher than the corresponding transcript ratios.

Gas1p, Gas3p and Gas5p are classified in glycoside hydrolase family 72, and are thought to be directly involved in β-1,3-glucan remodeling (Popolo & Vai, 1999; Mouyna *et al.*, 2000). In *gas1*Δ cell walls, a considerable amount of β-1,3-glucan is present (Ram *et al.*, 1998). This raises the question of whether, in a *gas1*Δ mutant, another member of this family may compensate for the loss of Gas1p function in β-1,3-glucan remodeling. Our data suggest that Gas5p but not Gas3p may become more relevant, as in *gas1*Δ cells three times more Gas5p is incorporated into the cell wall. Our observation is in agreement with the results of Schuldiner *et al.* (2005), who found a genetic interaction between *GAS1* and *GAS5* by epistatic miniarray profiling. Interestingly, a search for suppressor genes of the synthetic lethality of a *kex2*Δ*gas1*Δ mutant revealed that increased expression of *GAS5* was able to rescue the mutant (Tomishige *et al.*, 2005). A recent *in vitro* study also showed that Gas5p has β-1,3-transglucosidase activity similar to Gas1p, whereas a similar activity could not be detected for Gas3p under the experimental conditions used (Ragni *et al.*, 2007).

Pst1p was here for the first time identified as an authentic covalently linked cell wall protein, probably because its strong upregulation in *gas1*Δ cells allowed its identification by MS. The increased level of Pst1p in the wall is also consistent with the increased mRNA ratio of 3.87 observed by Lagorce *et al.* (2003). The level of wall-bound Ecm33p, which is a homolog of Pst1p, is also strongly increased in *gas1*Δ cells. This raises the question of whether this protein, which is normally mainly targeted to the plasma membrane, might now function in directly counteracting cell wall damage. Interestingly, similar to deletion of *GAS1*, deletion of *ECM33* is known to result in strong hypersensitivity to the cell wall perturbant Calcofluor White, swollen round cells, and an increased amount of β-1,6-glucosylated proteins being secreted into the growth medium (De Groot *et al.* 2001; Pardo *et al.*, 2004), suggesting that their functions might be related.

Crh1p and Crh2p belong to glycoside hydrolase family 16. Their role in cell wall biogenesis has been implicated in linking chitin to the β-1,6-glucan network (Rodríquez-Peña *et al.*, 2000; Cabib *et al.*, 2007). In the *gas1*Δ cell wall, β-1,3-glucan levels detected in the alkali-resistant fraction were reduced, although the chitin level was increased as compared to the wild type (Ram *et al.*, 1998). The insolubility of β-1,3-glucan in alkali is due to its linkage to chitin. With the limited availability of β-1,3-glucan, there seems to be an increased demand for enzymes that can crosslink chitins to β-1,6-glucan (Kapteyn *et al.*, 1997). This explanation is in agreement with our observation that the levels of both Crh1p and Crh2p have increased about fourfold.

## Statement

Re-use of this article is permitted in accordance with the Creative Commons Deed, Attribution 2.5, which does not permit commercial exploitation.
